# Internal (His)_6_-tagging delivers a fully functional hetero-oligomeric class II chaperonin in high yield

**DOI:** 10.1038/srep20696

**Published:** 2016-02-09

**Authors:** Danielle M. Paul, Fabienne Beuron, Richard B. Sessions, Andrea Brancaccio, Maria Giulia Bigotti

**Affiliations:** 1 School of Physiology, Pharmacology and Neuroscience, University of Bristol, Bristol BS8 1TD, UK; 2 Division of Structural Biology, The Institute of Cancer Research, London SW7 3RP, UK; 3 School of Biochemistry, University of Bristol, Bristol BS8 1TD, UK; 4 Istituto di Chimica del Riconoscimento Molecolare, CNR c/o Istituto di Biochimica e Biochimica Clinica, Università Cattolica del Sacro Cuore, 00168 Roma, Italy

## Abstract

Group II chaperonins are ATP-ases indispensable for the folding of many proteins that play a crucial role in Archaea and Eukarya. They display a conserved two-ringed assembly enclosing an internal chamber where newly translated or misfolded polypeptides can fold to their native structure. They are mainly hexadecamers, with each eight-membered ring composed of one or two (in Archaea) or eight (in Eukarya) different subunits. A major recurring problem within group II chaperonin research, especially with the hetero-oligomeric forms, is to establish an efficient recombinant system for the expression of large amounts of wild-type as well as mutated variants. Herein we show how we can produce, in *E. coli* cells, unprecedented amounts of correctly assembled and active αβ-thermosome, the class II chaperonin from *Thermoplasma acidophilum,* by introducing a (His)_6_-tag within a loop in the α subunit of the complex. The specific location was identified via a rational approach and proved not to disturb the structure of the chaperonin, as demonstrated by size-exclusion chromatography, native gel electrophoresis and electron microscopy. Likewise, the tagged protein showed an ATP-ase activity and an ability to refold substrates identical to the wild type. This tagging strategy might be employed for the overexpression of other recombinant chaperonins.

Chaperonins are large oligomeric protein complexes that play a crucial role in intracellular assisted protein folding. Ubiquitous in nature and indispensable for life, they are well conserved through all phyla^1^, wherein they are classified into two groups: type I chaperonins are those found in eubacteria and in eukaryotic organelles, while type II are typically found in Archaea and in the eukaryotic cytosol^2^,^3^. Chaperonins from the two groups share the same overall structure, in which 2 rings, composed of 7 (group I) or 8–9 (group II) subunits, are stacked back to back to form a toroidal cylinder enclosing a central cavity that provides a protected environment for non-native protein to fold in an ATP-dependent fashion without becoming enmeshed with other unfolded chains in the cell^4^,^5^. While the type I chaperonins, exemplified by the *E. coli* GroE system (GroEL and its co-chaperonin GroES), are reasonably well understood, the group II complexes are more complicated and more challenging to study, mainly due to their higher degree of structural complexity, which has been and still is the object of extensive investigation by electron microscopy and X-ray crystallography^5^,^6^,^7^. The composition in subunits of group II chaperonins does indeed range from that of the homo-oligomers and αβ hetero-oligomers found in Archaebacteria to that of the hetero-oligomers of 8 different subunits (catalogued from α to θ or 1 to 8) found in Eukarya, where the complex is defined as CCT/TriC (for chaperonin containing TCP-1/TCP-1 ring complex).

Such heterogeneity in subunits makes the recombinant expression of correctly assembled, fully functional hetero-oligomeric complexes particularly challenging. A great deal of work has been already dedicated to devise original recombinant strategies for tagging group II subunits in order to efficiently recover quantitative amounts of the entire complex from yeast cells or upon recombinant expression in bacterial and, more recently, mammalian cells^8^,^9^,^10^. In *S. cerevisiae*, an internal tag was introduced (within a loop) in the apical domain of CCT3/γ, which allowed the recovery of the whole complex through affinity purification^8^, whereas the CCT4 and CCT5 human subunits have also been tagged and expressed recombinantly in *E. coli*, and shown to be able to form active hexadecamers, although in a homo-oligomeric form^9^. On the other hand, fully assembled hetero-oligomeric complexes have been obtained in mammalian cells by expressing human CCT with an affinity tag on subunit 1, but the protein yield has proved to be very low^10^.

In general, a system for the efficient expression and purification of type II hetero-oligomeric chaperonins in *E. coli* giving large amounts of genetically modifiable complexes, would provide an invaluable contribution to the study and comprehension of these fascinating molecular motors. Hence, there is a strong interest in developing such recombinant expression systems, both in terms of the final protein yield achieved and of the rapidity of the process.

In previous work we presented the first expression system for the production in *E. coli* of homogeneous, fully functional αβ-hecadecamers of the thermosome from *Thermoplasma acidophilum*^11^, an archaeal chaperonin composed of two rings of alternating α and β subunits whose structure has been solved at high resolution^5^. We were able to clone in tandem and express in *E. coli* the genes encoding for the two subunits, obtaining reasonable amounts of correctly assembled and active complex. Here we report the introduction of six histidines ((His)_6_-tag) within a loop of the α-subunit, whose suitability was designed and modelled on the basis of structural and functional considerations, allowing the production of unprecedented amounts of functional αβ-thermosome hexadecamers. The structural and functional similarity of this recombinant product to the wild type αβ-thermosome is demonstrated by analytical methods, negative staining and cryo-electron microscopy and by the analysis of the refolding of endogenous substrates.

## Results

### (His)_6_-tagging of the α subunit

**Figure 1:**
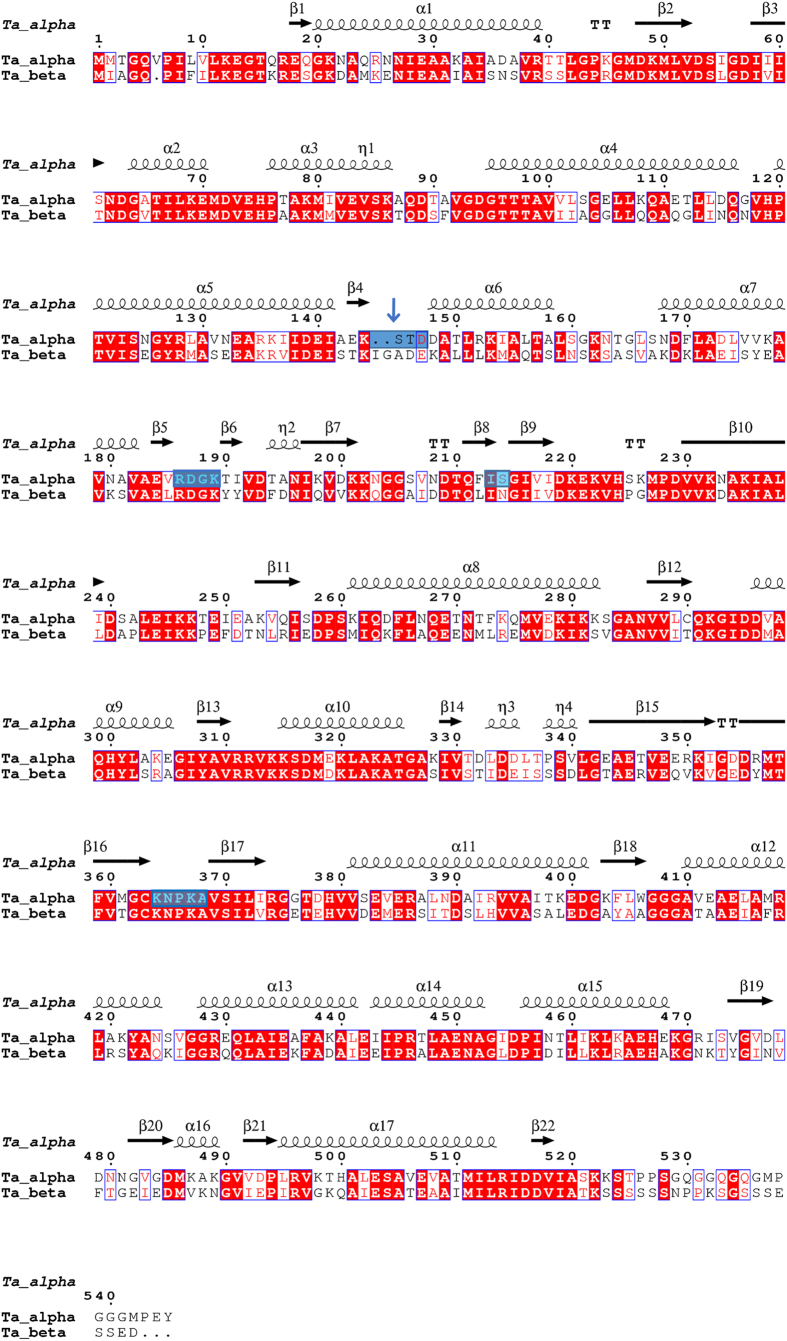
Planned locations for the insertion of an internal (His)_6_-tag in the thermosome from *T. acidophilum*. The candidate loops, identified from the 3D-structure, are highlighted by the blue boxes over the sequence of the α subunit aligned with the β-subunit. Red boxes identify the regions of sequence identity and the open boxes those of sequence similarity. The arrow shows the loop chosen and the low sequence similarity between the two subunits in this loop is evident. The elements of secondary structure are also reported on top of the corresponding amino acid sequence.

**Figure 2:**
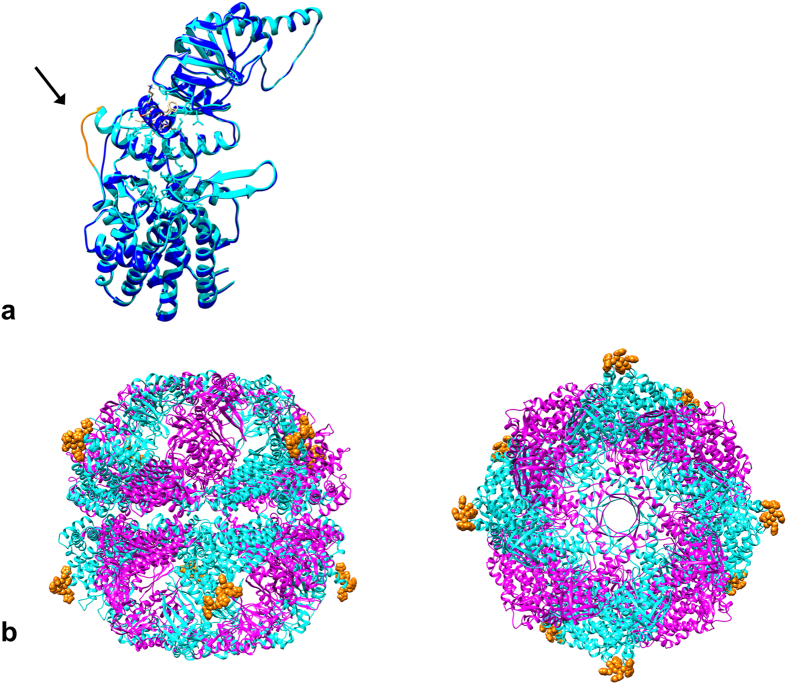
General architecture of the (His)_6_-tagged thermosome. (**a**) Schematic drawing of the secondary structural elements of the thermosome α-subunit in the absence (blue) and in the presence (cyan) of a (His)_6_-tag (gold, and indicated by the arrow) inserted in an exposed loop between the equatorial and the intermediate domain. (**b**) Side view (left) and top view (right) of the hexadecameric thermosome based on the crystallographic structure^5^, with the (His)_6_-tags (gold) modelled in the 8 α-subunits (cyan), exposed on the surface of the complex. The un-tagged β-subunits are shown in magenta.

While the expression and purification process described in Bigotti and Clarke (2005) (giving 2–4 mgs of pure α_8_/β_8_ thermosome/liter of culture) is efficient, it is still rather time-consuming and prone to variability in the yield of pure protein. Hence we sought to optimize the process by inserting a tag in one of the subunits for affinity purification. We chose the α subunit based on two pieces of evidence^11^. Firstly, the β subunit is more unstable and prone to the action of proteases. Secondly, unlike the β subunit alone, the α subunit alone can self-assemble in stable α_16_ complexes, allowing the isolation of the homo-oligomeric complex from cells expressing only the α subunit. Unfortunately both the C- and N-termini of the subunit are located internally, in a region of the equatorial domain facing the inner chamber of the thermosome, so that tags attached to the chain termini are poorly accessible and result in inefficient affinity purification^12^. By analyzing the structure of the thermosome (PDB id: 1A6D), a series of surface-exposed loops were identified that could accommodate an internal tag, possibly without interfering either with the assembly of the complex or with its strongly allosteric behavior. Figure 1 shows the locations of loops that are candidates for insertion of a (His)_6_-tag mapped onto a sequence alignment of the α and β subunits annotated with the secondary structure. It is worth mentioning that the loop between beta sheets β5–β6 has already been chosen for a (His)_6_-tag insertion used in the production of the α_16_ complex^13^ for structural studies. We judged this location to be too close to the α/β subunit interface to be optimally placed. Our first-choice location is between beta sheet β4 and alpha helix α6 since this appeared to be an ideal position just at the base of the intermediate, hinge domain that follows the equatorial, ATP binding domain. Panel a of Fig. 2 is a superimposition of the thermosome α subunit without (brilliant blue) and with (light blue) (His)_6_-tag inserted in this loop after Lys144 and highlighted by the arrow; the fully assembled thermosome thus presents eight His-tags on its surface, four on each ring (Fig. 2b), providing protruding anchors for immobilization on Ni^2+^resin.

### Cloning, expression and purification of the His-tagged α_8_/β_8_ thermosome

**Figure 3:**
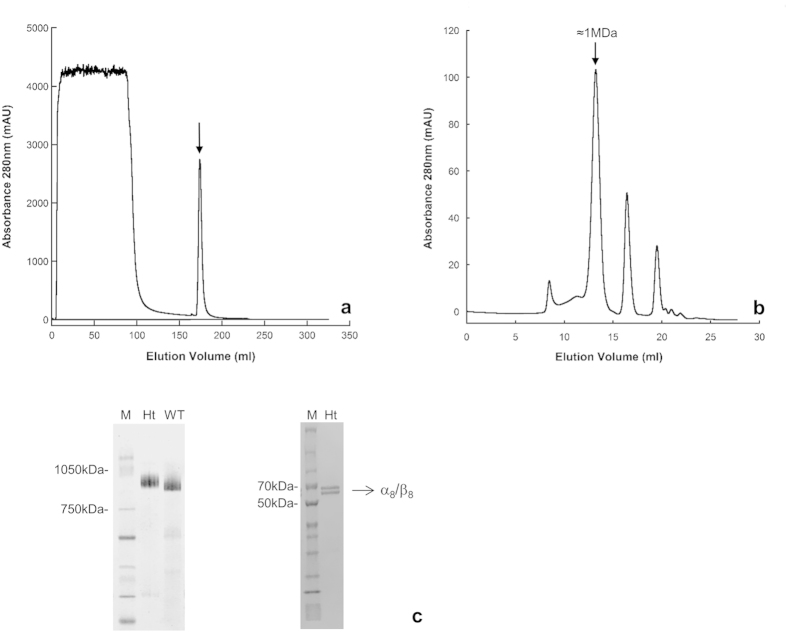
Two-step purification and electrophoretic analysis of the α_8_/β_8_ (His)_6_-tagged complex. (**a**) First purification step: chromatogram of the *E. coli* cell lysate run on a Nickel affinity column. The arrow indicates the sharp peak of eluate containing the tagged thermosome. (**b**) Second purification step: the peak eluted from the affinity column is subjected to size exclusion chromatography (SEC) on a Superose 6 column, which results in the separation of a peak, highlighted by the arrow, with a retention time indicative of a molecular mass ≈1 MDa, characteristic of a hexadecameric complex. Further analysis of this peak via native gel (panel c, left) reveals the presence of a single band of the expected molecular mass, with an electrophoretic mobility slightly slower than that of the un-tagged thermosome, as expected given the contribution of 8×(His)_6_ per complex. Finally, SDS-PAGE of the same sample (panel c, right) clearly separates two bands of ≈60 kDA, corresponding to the α- and β-subunits, in a 1:1 ratio, confirming that the species isolated by SEC is the α_8_/β_8_ (His)_6_-tagged complex. Ht: (His)_6_-tagged thermosome, WT: untagged recombinant thermosome, M: molecular weight marker.

A synthetic gene for the α subunit with a (His)_6_-tag downstream Lys144 (αK144Ht) was used to replace the wild-type α gene in the pETTherm α/β construct previously described^11^ for expression of the tagged thermosome in *E. coli* cells. Similarly to the wild-type construct, the overall expression level for both subunits was 15–20% of the soluble proteins, but a much higher percentage of those (about 50%) were found correctly assembled in α/β hexadecamers at the end of the two-step purification, as opposed to the relatively small fraction of subunits that were found to form the un-tagged α_8_/β_8_ thermosome after a multi-step purification procedure (≤15% of the initial population). Figure 3 illustrates the purification process, with a first step of affinity chromatography (panel a) that selects the (His)_6_-tag containing species, and a second one that isolates the ≈960 kDa complex (panel b) by size-exclusion chromatography (SEC). The purity and identity of the high molecular weight species eluted as a single, sharp peak from SEC were checked by native gel electrophoresis (panel c), which resulted in a single, high molecular weight band running slightly slower than the un-tagged thermosome, and by SDS-PAGE (panel d), that shows two clear bands corresponding to the α and β subunits, in a 1:1 ratio, with no detectable impurities. Taken together, these results are strongly indicative of a population of α_8_/β_8_ thermosome purified to homogeneity. It is worth noting that a further, high resolution SEC on a medium for the separation of lower molecular weight species (i.e. Sephadex 200) run in the presence of 20% methanol, allowed for the isolation of small hydrophobic contaminants, but only in traces amounts, thus revealing this further step to be unnecessary. The average time required for the whole purification procedure is 48 hours, the final yield of the protein obtained is ≈10 mg/liter of bacterial culture and the product remains stable for days at a time when kept at 4 °C.

### Negative staining and cryo-electron microscopy

**Figure 4:**
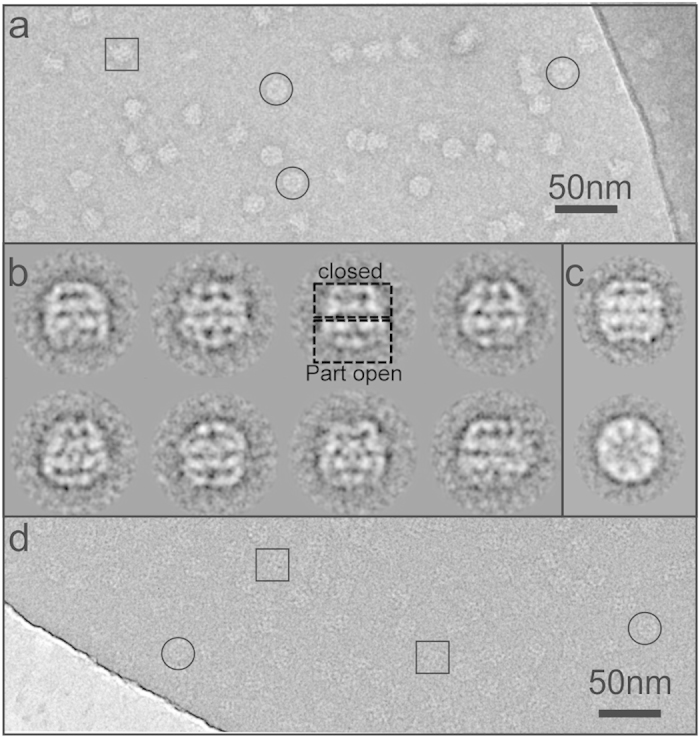
Electron microscopy analysis. (**a**) Micrograph of negatively stained thermosome. The particles appear randomly oriented on the grid with a predominance of side views (≈93%); top views are highlighted by black circles. An example of a more open conformation is identifiable in the sample (black square). (**b**) Selection of 2D class averages from the negative stain EM data set which indicate that the complex adopts a predominantly ‘partially open-closed’ conformation (dashed boxes). Each class average is composed of 10–20 particle images. **(c**) The closed conformation is also visible in the 2D class averages from cryo-EM data (side and top view). (**d**) Cryo-EM experiments led to randomly oriented particles with a similar proportion of side-views to the negatively stained data set. The black circles and squares identify top views and side views, respectively.

The identity and assembly state of the tagged complex was confirmed by electron microscopy. The initial appearance of the negatively stained particles on the grid (Fig. 4a) indicated that there was a predominance of side-on views of the complex. Only ~7% of the ≈13,000 particle data set were top views. Side-on views provide a higher degree of structural information in 2D projection and aid in the identification of the conformation of the complex. Upon inspection of the 2D class averages, a large population is identifiable in which one of the rings is closed, while the other appears more open, with clear gaps between the apical domains (Fig. 4b). We would describe this as a ‘partially open-closed’ conformation. The 2D averages are asymmetric and resemble the closed-open (bullet) form described for the α_16_ thermosome^14^, but appear to be more closed than the typical ‘bullet’ shape described for the partially-closed state found in the archaeal group II chaperonin from *Methanococcus maripaludis*^15^. Class averages of the fully closed complex (Fig. 4c) could be identified by their more square shape when viewed from the side (upper panel). The top view average (Fig. 4c, lower panel) shows that the rings are formed of 8 subunits and exhibits a diameter of 160 Å, in agreement with the crystal structure^5^. The complex observed in an apo-state (i.e. in the absence of nucleotides) by cryo-electron microscopy also displayed the same conformations described in the negatively stained data (Fig. 4d).

### Steady-state ATPase activity and refolding of endogenous substrates

**Figure 5:**
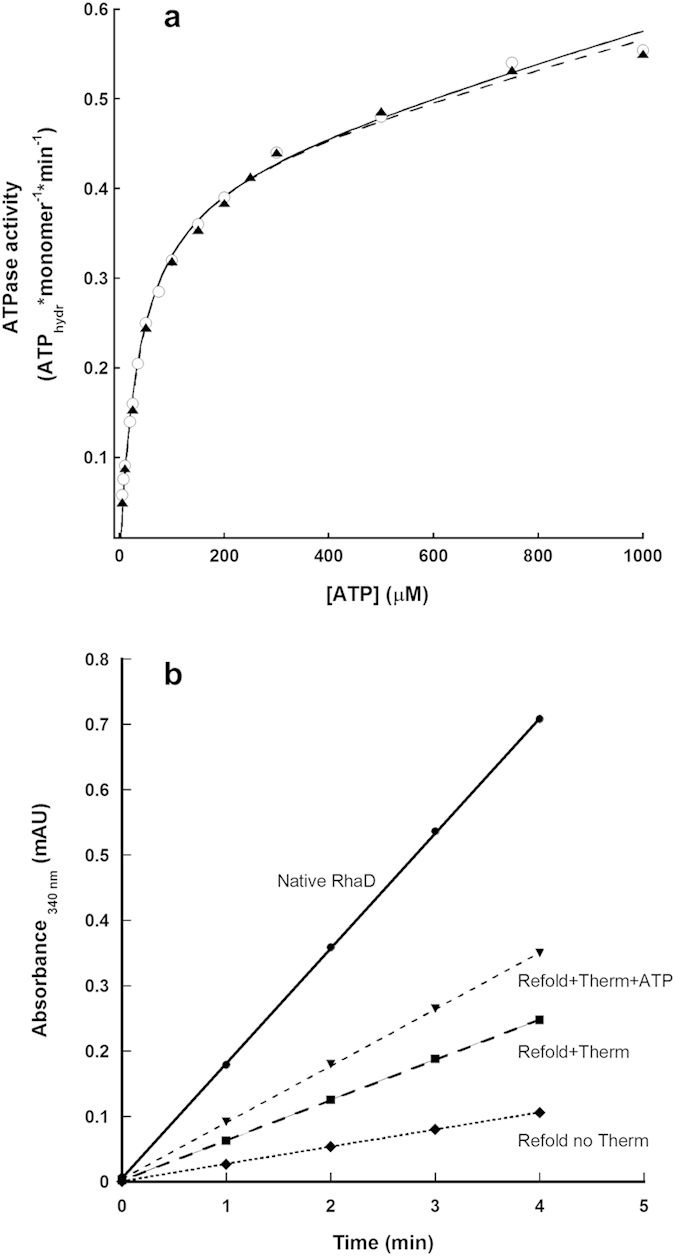
ATP hydrolysis and substrate refolding activities of the α_8_/β_8_ His-tagged thermosome at 55 °C. (**a**) The ATPase activity of the α_8_/β_8_ (His)_6_-tagged complex as a function of ATP concentration is reported for the tagged thermosome (closed triangles) and for the un-tagged one (open circles); the fits of the two sets of data to a sum of tight and weak binding sites (see both Results and Materials and Methods for details) are represented by the dashed and the continuous lines, respectively. (**b**) Substrate refolding activity of the (His)_6_-tagged thermosome. The recovery in activity (indicating the refolding yield) of chemically denatured Rhamnose dehydrogenase from *T. acidophilum* upon 50× dilution in buffer in the different conditions reported, was measured as an increase in absorbance at 340 nm (see Materials and Methods). The lag times do not change significantly in the different conditions, and have been removed. The tagged thermosome increases the refolding yield of the endogenous substrate to ≈50% in the presence of ATP (see Table 1). Each data point shown is the average of at least three independent experiments.

The hydrolytic activity of the (His)_6_-tagged thermosome as a function of ATP concentration is shown in Fig. 5a. These data were measured at the physiological temperature of 55 °C using the coupled enzymatic essay described in Materials and Methods. The curve shape is indistinguishable from that of the un-tagged recombinant system (also reported on the plot) and of the native chaperonin^16^ and shows two phases, indicating the presence of two different binding sites, with K_M1_ = 15 μM ATP for the tighter and K_M2_ = 370 μM ATP for the weaker site. As already discussed for the un-tagged complex^11^, this could depend either on negative cooperativity between rings (so that at low ATP concentrations only one ring is active, as also shown for mammalian CCT^17^) or on a different behaviour of the α and β subunits, that would mirror the case of eukaryotic chaperonins^18^, or on both. As expected based on the striking similarity with the un-tagged thermosome, the rates of ATP turnover are slow, with values of 2.15 moles/(mol ring) per minute for the tighter sites and of 3.2 moles/(mol ring) per minute for the weaker.

In order to measure the effect of the tagged thermosome on protein folding, we first identified a series of enzymes from *T. acidophilum* whose activity could be easily recorded spectrophotometrically to use as possible substrates in refolding experiments and, where not available otherwise, we cloned and expressed them in *E.coli* (see Materials and Methods for details). In particular, two dehydrogenases with peak activities at 55 °C have been used in the experiments described herein: aldohexose dehydrogenase (AldT)^19^, catalysing the NAD^+^ dependent oxidation of D-mannose, and rhamnose dehydrogenase (RhaD)^20^, which catalyses the oxidation of L-rhamnose in the presence of NADP^+^. The proteins were chemically denatured in 6 M guanidine hydrochloride and the refolding reaction was started by massive dilution into buffer either in the presence or absence of untagged thermosome, with or without ATP added. The regain in activity of the unfolded enzymes upon dilution at 55 °C was monitored by the rate of increase in absorbance at 340 nm corresponding to the formation of NADH/NADPH. This assay was used as a direct indicator of the refolding yield in the different conditions by comparison with the activity of the pure enzymes. The results obtained with the two substrates were comparable and the refolding yields are summarised in Table 1, and data for RhaD are shown graphically in Fig. 5b. The substrate shows a certain degree of spontaneous refolding, and when an excess of thermosome is added to the dilution mixture the refolding yield doubles, with a recovery of ~35% of the original activity, reaching values around 50% in the presence of ATP. Negative controls in which the thermosome was substituted by a thermophilic protein (pyruvate kinase from *B. stearothermophilus*) did not exhibit any effect on the refolding yield (see Table 1). These results show that the tagged thermosome strongly promotes folding of endogenous substrates, even in the absence of ATP. It should be noted that this latter behaviour does not depend on the His-tagging of the chaperonin, as confirmed by identical (within the experimental error) results obtained performing these experiments with the un-tagged thermosome (data not shown). The lag phase of the recovery in activity (not reported in the graph for clarity) is typically between 1 and 3 minutes and was not significantly affected by the presence of thermosome, either with or without ATP.

## Discussion

Since their discovery in the mid 90’ s, much effort has been invested in producing efficient recombinant systems for structural and functional studies on class II chaperonins^8^,^9^,^10^,^11^,^12^,^13^, one of the main challenges being the difficulty in obtaining homogeneous populations of correctly assembled complexes, a difficulty that increases proportionally with the degree of subunit heterogeneity. The possibility of expressing isolated subunits recombinantly in *E. coli* cells and reconstituting them into structurally and functionally viable complexes *in vitro* is certainly very appealing, but this approach has mainly been successful in obtaining homo-oligomeric variants. We tackled the problem and produced the first recombinant hetero-oligomeric model system by cloning in tandem in a single construct the α and β subunits of the thermosome from *T. acidophilum* and expressing them in *E.coli* in a correct hexadecameric form^11^. However, as reported for other recombinant systems, our typical recurring problem has been the low yield of pure active protein after a time-consuming purification protocol requiring the use of multiple chromatographic steps over a period of several days, with an ensuing loss of protein in an unpredictable fashion due to the complexity of the overall procedure.

This issue prompted us to explore improving the efficiency of the recombinant expression system by introducing a short linear tag into one of the subunits to facilitate the purification procedure and increase the final yield of protein. We used a rational approach based on the known 3D-structure to identify surface loops in which the shortest possible affinity tag, a (His)_6_-tag, could be introduced without interfering with the local tertiary structure of the subunit, the overall assembly of the double-ringed structure and without adversely affecting the complex network of interactions underlying the chaperonin’s allosteric behaviour. The first choice among the loops identified was based on low sequence similarity between the α- and β-subunits and on its location between the equatorial and the intermediate domains as well as remoteness from the α and β subunit interface. Inserting the (His)_6_-tag into this loop proved to be ideal for our purposes, allowing us to shorten and improve the purification protocol, with a final yield of intact α_8_/β_8_ thermosome about 2.5 times higher than that previously reported^11^. The insertion of 4×(His)_6_-tags per ring does not interfere with the assembly and the overall structure of the thermosome, as shown by analytical techniques such as SEC and gel electrophoresis, both in native and denaturing conditions, and by negative staining and cryo- EM, whose 2D class averages (Fig. 4b,c) reveal the presence of particles with a clear 8-fold symmetry, composed of two rings with an estimated size which is typical of the thermosome. The apo-form presents a certain degree of conformational heterogeneity under the experimental setup here described, and the preponderant species is the partially open-closed complex. This is confirmed by the cryo-EM data collected (see raw images in Fig. 4d). It has to be noted that the resolution of the EM results presented here does not allow the determination of the specific arrangement of subunits in the complex, but there are two observations that strongly indicate it to be composed of 2×(αβ)_4_ rings. First of all, the assembled complex behaves like the native thermosome with respect to the binding and hydrolysis of ATP at the physiological temperature of *T. acidophilum*. Second, before cloning them in tandem in the same vector, we have expressed the α and β subunits separately. Although the α_16_ species is stable, the β subunit cannot assemble into single or double-ring structures, making it unlikely that an α_8_ ring would associate very effectively with a β_8_ ring to produce an α_8_/β_8_ structure in which each ring is homo-oligomeric.

Moreover, the (His)_6_ tags protruding on the surface of this recombinant chaperonin could represent an important advantage for the alignment of the particles and the discrimination of the different subunits, which is one of the main problems in the structure determination of hetero-oligomeric chaperonins by EM. Indeed, one of the ways to overcome such difficulties in the eukaryotic chaperonin has been to bind antibodies to the complex raised against two specific subunits^21^, and a similar approach could be applied to the tagged thermosome, where the α-subunit could be labelled with anti-His antibodies^22^ or with their Fabs^23^, as to reduce the size of the protruding molecules. This would allow in the first instance to determine by cryo-EM the arrangement of the α and β subunits in each ring, and exclude any formally possible arrangements other than 2×(αβ)_4_. In a more general picture, the decoration with external His-tags may become a powerful tool for several applications, not necessarily limited to EM analysis.

The activity of the recombinant thermosome is unaffected by the insertion of the (His)_6_-tag. The ATPase behaviour of the tagged complex is indistinguishable from the un-tagged one (Fig. 5a), and it is active in refolding unfolded polypeptides. The refolding activity has been demonstrated here for the first time on endogenous putative substrates. All these characteristics lead us to believe we have finally established a state-of-the-art system for the study of the thermosome. The fast and efficient production of specific variants will allow the characterisation of the mechanism of action of this protein and give us the opportunity to shed light onto the differential behaviour of the two subunits. This will not only reveal how this complex machinery works, but would be a fundamental step in understanding why the structural heterogeneity of class II chaperonins has evolved from archaea (homo- and hetero-oligomeric complexes) to eukaryotes (oligomers of eight different subunits with different affinities for both ATP and substrates^18^,^24^). In this respect, the recombinant system hereby described could also be used in the construction of ‘synthetic’ chaperonins using different eukaryotic subunits, alone and in well determined couples, as a mean for studying their specific characteristics and behaviour and analysing their reciprocal interactions.

Furthermore, a neurological disorder has recently been found to depend on a point-mutation in the mammalian chaperonin^25^. The effect of this mutation has been assessed by introducing it into the framework of a homo-oligomeric chaperonin from *Pyrococcus furiosus*, where it reduces the stability of the double-ringed complex^26^. In light of these results, our system may also represent an interesting venue to study the mechanisms behind the pathological consequences caused by deleterious point mutations found in eukaryotic group II chaperonins.

## Methods

### (His)_6_-tagging of the α subunit

The exposed loops for internal (His)_6_ tagging were located analyzing the α-subunit in the context of the high resolution structure available^5^ (PDB code: 1A6D).

The sequence alignment of the thermosome subunits (accession codes: α, P48424 and β, P48425) was performed in MUSCLE 3.8^27^ via the resources of EMBL/EBI (http://www.ebi.ac.uk/Tools/msa). The illustration of multiple sequence alignment including the secondary structure elements was generated via ESPript 3.0 (http://espript.ibcp.fr/ESPript/ESPript/)^28^, using the PDB accession code 1A6D.

The structural model of the α-subunit with a (His)_6_ inserted downstream Lys144 was constructed using the loop building module of InsightII (2005, Accelrys).

### Cloning, expression and purification of the His-tagged α_8_/β_8_ thermosome

The synthetic gene of the K144-(His)_6_ α-subunit, αK144Ht (Invitrogen), was used to replace by ‘cut and paste’ (5′ ends: NdeI, 3′ ends: EcoRI) the wild-type gene in the construct pETTherm α/β for the expression of the un-tagged α_8_/β_8_ thermosome as elsewhere described^11^. The new construct thus obtained, αK144HTTherm, was used to transform chemically competent *E. coli* BL21(DE3) Codon Plus-RIL cells; a single transformed colony was grown in LB medium plus kanamycin (Km, 30 μg/ml) in an orbital shaker at 37 °C overnight and, after refreshing by a 100× dilution in LB plus Km, when the culture reached an OD_600_ ≈0.8 expression was induced by adding 0.7 mM IPTG. Cells were collected after 5 hours by centrifugation, and the pellet was resuspended in binding buffer (20 mM NaPho, 0.5 M NaCl, 20 mM imidazole, pH 7.2) with added benzonase and EDTA-free inhibitors cocktail (both from Sigma). The cells were lysed in a cell disruptor (Constant Systems, model Z plus 1.1 KW), the cell debris was spin down by centrifugation and the supernatant was loaded onto a 5 ml HisTrap FF column (GE Healthcare) equilibrated in binding buffer. This and the following chromatographic purification step were carried out at room temperature on an ÄKTA purifier 900 system, run by the UNICORN 4.0 software (Amersham). After extensive washing with binding buffer, elution with an imidazole gradient between 20 and 500 mM resulted in a single sharp peak eluting at an imidazole concentration ≈100 mM. Fractions corresponding to this peak were run on SDS-PAGE and those resulted to contain the bands corresponding to the α- and β-subunits in a 1:1 ratio were pooled, extensively dialysed in buffer A (25 mM TrisHCl, 150 mM NaCl, 5 mM EDTA pH 7.5), concentrated by ultrafiltration in Vivaspin20 filter units (Vivascience) if necessary and loaded on a gel filtration column (depending on the volume to be loaded, either on a HiLoad 26/600 Superdex 200 or on a Superose 6 Increase 10/300 GL, both from GE Healthcare). The high molecular weight fractions found by SDS-PAGE to contain only the α- and β-subunits in a 1:1 ratio with no detectable contaminants were pooled, concentrated by ultrafiltration in Vivaspin20 filter units (Vivascience) and, after the addition of 15% glycerol, snap frozen for conservation at - 80°C. The pure protein after thawing was shown to be stable at 4 °C for at least one week. Protein concentration was determined spectrophotometrically as described^11^.

All the enzymes used for nucleic acid manipulation were either from New England Biolabs or Roche, and the kits used for DNA prepping and extraction/purification where from Qiagen.

### Negative staining and cryo-electron mycroscopy

#### Sample preparation

Negative staining: The sample was applied onto a carbon film supported by R2/2 Quantifoil electron microscope grids previously rendered hydrophilic by glow discharging in air. The sample was then negatively stained using 2% Uranyl acetate. Images were collected on a FEI Tecnai F20 electron microscope operated at an accelerating voltage of 200 kV and with a 4K × 4K F416 TVIPS CMOS detector at a nominal magnification of 29 K, resulting in a sampling of 2.892Å per pixel. The grids were imaged under low-dose conditions and 132 frames were recorded with EM-Tools automated image acquisition software.

Cryo-EM: The sample was applied onto a thin layer of carbon freshly floated from mica and supported by R1.2/1.3 Quantifoil grids and flash frozen in ethane using an FEI vitrobot Mark III, at 95% humidity and 19 °C, and imaged as above.

#### Image processing

Particles were picked using e2boxer.py as part of the EMAN2 single particle image processing software suite^29^. The particles were extracted into 128 × 128 boxes normalised and band-pass filtered between 200 and 10 Å using Spider routines^30^. The negative stain data set comprised of ≈13 K particles was submitted to the ISAC classification procedure in SPARX^31^.

### Steady-state ATPase activity

All the experiments were conducted at 55 °C as described for the un-tagged thermosome^11^, as was the data fitting (see therein for the definition of the combined equation used).

### Refolding of endogenous substrates

A series of possible substrates was identified in *T. acidophilum* whose recovery in activity during refolding experiments could be assessed spectrophotometrically, and those not available otherwise were cloned and expressed in *E. coli*. Of the two enzymes used for the refolding experiments herein reported, the construct for AldT recombinant expression in *E. coli* was a kind gift from Prof. Tomohiro Tamura (AIST, Sapporo, Japan); the enzyme was overexpressed and purified as described^32^. Whereas the synthetic gene for RhaD recombinant expression was obtained from Invitrogen and inserted in the polylinker of pET15b, between the restriction sites for XhoI (5′end) and BamHI (3′end), for expression fused with a (His)_6_-tag at the N-terminus. The construct pET15RhaD thus obtained was used to transform chemically competent *E. coli* BL21(DE3) Codon Plus-RIL cells; a single transformed colony was grown in LB medium plus carbenicillin (Carb, 100 μg/ml) in an orbital shaker at 37 °C overnight and, after refreshing by a 100× dilution in LB plus Carb, when the culture reached an OD_600_ ≈ 0.8 it was induced by adding 1 mM IPTG. Cells were collected after 3 hours by centrifugation, and the pellet was resuspended in binding buffer (20 mM NaPho, 0.5 M NaCl, 20 mM imidazole, pH 7.2) with added benzonase and EDTA-free inhibitors cocktail (both from Sigma). The cells were lysed in a cell disruptor (Constant Systems, model Z plus 1.1 KW), the cell debris was spin down by centrifugation and the supernatant was loaded onto a 5 ml HisTrap FF column (GE Healthcare) equilibrated in binding buffer. Affinity chromatography was carried out at room temperature on an ÄKTA purifier 900 system, run by the UNICORN 4.0 software (Amersham). After extensive washing with binding buffer, elution with an imidazole gradient between 20 and 500 mM resulted in a single peak eluting between 150 and 300 mM imidazole. Fractions corresponding to this peak were run on SDS-PAGE, and those migrating as a single band of ≈35 KDa (the molecular mass of RhaD) with no detectable contaminants were pooled and extensively dialysed in 20 mM TrisHCl, 0.1 M NaCl, 2 mM EDTA, 12% glycerol, pH7.7. The activity of the recombinant enzyme at 55 °C was the same as that of native RhaD^20^. Protein concentration was determined spectrophotometrically with extinction coefficients at 280 nm estimated to be, based on the protein sequence, 26.000 M^−1^cm^−1^ for AldT and 17.420 M^−1^cm^−1^ for RhaD. All the reagents used in the activity assays were of analytical grade and purchased from Sigma.

The renaturation assays were initiated by a 50-fold dilution of 1 μM denatured (in 6 M guanidinium hydrochloride) AldT or RhaD in the standard chaperonin reaction buffer (25 mM TrisHCl, 50 mM KCl, 20 mM MgCl_2_ and 50 mM NaCl, pH 7.5.) containing 0.5 mM NAD^+^ and the substrate D-mannose (1 mM), or 1 mM NADP^+^and the substrate L-rhamnose (1 mM), respectively. When required, the thermosome (1 μM complex) was added to the refolding buffer before dilution of the substrate. The recovery in activity of the substrate enzymes was measured spectrophotometrically as an increase in the absorbance at 340 nm following the reduction of either NAD^+^ or NADP^+^, resulting from the conversion of D-mannose or L-rhamnose (Sigma) to D-glucono-1,5-lactone and L-rhamnono-1,4-lactone, respectively. NAD^+^ and NADP^+^ were purchased from Sigma and Roche.

## Additional Information

**How to cite this article**: Paul, D. M. *et al.* Internal (His)_6_-tagging delivers a fully functional hetero-oligomeric class II chaperonin in high yield. *Sci. Rep.* **6**, 20696; doi: 10.1038/srep20696 (2016).

## Figures and Tables

**Table 1:** Effect of the (His)_6_-tagged thermosome on the refolding of *Thermoplasma acidophilum* rhamnose dehydrogenase (RhaD) and aldohexose dehydrogenase (AldT) at 55 °C.

	RhaD Activity (%) No ATP	RhaD Activity (%) (+1 mM ATP)	AldT Activity (%) No ATP	AldT Activity (%) (+1 mM ATP)
Native sub.	98.4 ± 1.6	97.8 ± 2.2	98.1 ± 1.9	99 ± 2.3
Unfolded sub.	14.8 ± 0.9	14.3 ± 0.8	13.2 ± 1.7	13.5 ± 2.1
Unfolded sub + PK	14.2 ± 1.1	15 ± 1.6	13.8 ± 1.8	14.3 ± 1.6
Unfolded sub + Ht th.	35.2 ± 2.8	49.5 ± 1.3	34 ± 2.4	47.6 ± 1.9

The refolding yield is expressed as recovery in steady state activity, relative to the activity of the native dehydrogenases, of the unfolded substrate after a 50× dilution in buffer in the conditions indicated. sub.: substrate, Ht th.: (His)_6_-tagged thermosome, PK: pyruvate kinase from *B. stearothermophilus*, used as control to exclude any non-specific effect of a thermophilic protein on the refolding yield.

## References

[b1] Skjærven L., Cuellar J., Martinez A. & Valpuesta J. M. Dynamics, flexibility, and allostery in molecular chaperonins. FEBS Lett. 10.1016/j.febslet.2015.06.019. [Epub ahead of print] (2015). 26140986

[b2] Bigotti M. G. & Clarke A. R. Chaperonins: The hunt for the Group II mechanism. Arch. Biochem. Biophys. 474, 331–339 (2008). 18395510 10.1016/j.abb.2008.03.015

[b3] Lopez T., Dalton K. & Frydman J. The mechanism and function of group II chaperonins. J. Mol. Biol. 10.1016/j.jmb.2015.04.013 (2015). PMC4706738 25936650

[b4] Braig K. *et al.* The crystal structure of the bacterial chaperonin GroEL at 2.8 Å. Nature 371, 578–586 (1994). 7935790 10.1038/371578a0

[b5] Ditzel L. *et al.* Crystal structure of the thermosome, the archaeal chaperonin and homolog of CCT. Cell 93, 125–138 (1998). 9546398 10.1016/s0092-8674(00)81152-6

[b6] Zhang J. *et al.* Mechanism of folding chamber closure in a group II chaperonin. Nature 463, 379–383 (2010). 20090755 10.1038/nature08701 PMC2834796

[b7] Kalisman N., Schröder G. F. & Levitt M. The crystal structures of the eukaryotic chaperonin CCT reveal its functional partitioning. Structure 21, 540–549 (2013). 23478063 10.1016/j.str.2013.01.017 PMC3622207

[b8] Pappenberger G., McCormack E. A. & Willison K. R. Quantitative actin folding reactions using yeast CCT purified via an internal tag in the CCT3/γ subunit. J. Mol. Biol. 360, 484–496 (2006). 16762366 10.1016/j.jmb.2006.05.003

[b9] Sergeeva O. A. *et al.* Human CCT4 and CCT5 chaperonin subunits expressed in *Escherichia coli* form biologically active homo-oligomers. J. Biol. Chem. 288, 17734–17744 (2013). 23612981 10.1074/jbc.M112.443929 PMC3682573

[b10] Machida K. *et al.* Reconstitution of the human chaperonin CCT by co-expression of the eight distinct subunits in mammalian cells. Protein Expr. Purif. 82, 61–69 (2012). 22133715 10.1016/j.pep.2011.11.010

[b11] Bigotti M. G. & Clarke A. R. Cooperativity in the thermosome. J. Mol. Biol. 348, 13–26 (2005). 15808850 10.1016/j.jmb.2005.01.066

[b12] Waldman T., Nitsch M., Klumpp M. & Baumeister W. Expression of an archaeal chaperonin in *E.coli*: formation of homo- (α,β) and hetero-oligomeric (α+β) thermosome complexes. FEBS Lett. 376, 67–73 (1995). 8521970 10.1016/0014-5793(95)01248-8

[b13] Nitsch M., Klumpp M., Lupas A. & Baumeister W. The thermosome: alternating α and β-subunits within the chaperonin of the archaeon *Thermoplasma acidophilum*. J. Mol. Biol. 267, 142–149 (1997). 9096213 10.1006/jmbi.1996.0849

[b14] Schoehn G., Hayes M., Cliff M., Clarke A. R. & Saibil H. R. Domain rotations between open, closed and bullet-shaped forms of the thermosome, an archaeal chaperonin. J. Mol. Biol. 301, 323–332 (2000). 10926512 10.1006/jmbi.2000.3952

[b15] Clare D. K. *et al.* Multiple states of a nucleotide-bound group 2 chaperonin. Structure 16, 528–534 (2008). 18400175 10.1016/j.str.2008.01.016 PMC2719814

[b16] Gutsche I., Mihalache O. & Baumeister W. ATPase cycle of an archaeal chaperonin. J. Mol. Biol. 300, 187–196 (2000). 10864508 10.1006/jmbi.2000.3833

[b17] Kafri G., Willison K. R. & Horovitz A. Nested allosteric interactions in the cytoplasmic chaperonin containing TCP-1. Protein Sci. 10, 445–449 (2001). 11266630 10.1110/ps.44401 PMC2373951

[b18] Reissmann S. *et al.* A gradient of ATP affinities generates an asymmetric power stroke driving the chaperonin TRIC/CCT folding cycle. Cell Rep. 2, 866–877 (2012). 23041314 10.1016/j.celrep.2012.08.036 PMC3543868

[b19] Yasutake Y., Nishiya Y., Tamura N. & Tamura T. Structural insights into unique substrate selectivity of *Thermoplasma acidophilum* D-aldohexose dehydrogenase. J. Mol. Biol. 367, 1034–1046 (2007). 17300803 10.1016/j.jmb.2007.01.029

[b20] Kim S. M., Paek K. H. & Lee S. B. Characterization of NADP^+^-specific L-rhamnose dehydrogenase from the thermoacidophilic archaeon *Thermoplasma acidophilum*. Extremophiles 16, 447–454 (2012). 22481639 10.1007/s00792-012-0444-1

[b21] Rivenzon-Segal D., Wolf S. G., Shimon L., Willison K. R. & Horovitz A. Sequential ATP-induced allosteric transitions of the cytoplasmic chaperonin containing TCP-1 revealed by EM analysis. Nat. Struct. Mol. Biol. 12, 233–237 (2005). 15696173 10.1038/nsmb901

[b22] Desfosses A., Goret G., Farias Estrozi L., Ruigrok R. W. & Gutsche I. Nucleoprotein-RNA orientation in the measles virus nucleocapsid by three-dimensional electron microscopy. J. Virol. 85, 1391–1395 (2011). 21106738 10.1128/JVI.01459-10 PMC3020520

[b23] Wąsowicz M., Milner M., Radecka D., Grzelak K. & Radecka H. Immunosensor incorporating anti-His (C-term) IgG F(ab’) fragments attached to gold nanorods for detection of His-tagged proteins in culture medium. Sensors (Basel) 10, 5409–5424 (2010). 22219669 10.3390/s100605409 PMC3247714

[b24] Joachimiak L. A., Walzthoeni T., Liu C. W., Aebersold R. & Frydman J. The structural basis of substrate recognition by the eukaryotic chaperonin TRiC/CCT. Cell 159, 1042–1055 (2014). 25416944 10.1016/j.cell.2014.10.042 PMC4298165

[b25] Sergeeva O. A., Tran M. T., Haase-Pettingell C. & King J. A. Biochemical characterization of mutants in chaperonin proteins CCT4 and CCT5 associated with hereditary sensory neuropathy. J. Biol. Chem. 289, 27470–27480 (2014). 25124038 10.1074/jbc.M114.576033 PMC4183788

[b26] Min W. *et al.* A human CCT5 gene mutation causing distal neuropathy impairs hexadecamer assembly in an archaeal model. Sci. Rep. 4 6688 (2014). 25345891 10.1038/srep06688 PMC4209464

[b27] Edgar R. C. MUSCLE: multiple sequence alignment with high accuracy and high throughput. Nucleic Acids Res. 32, 1792–1797 (2004). 15034147 10.1093/nar/gkh340 PMC390337

[b28] Robert X. & Gouet P. Deciphering key features in protein structures with the new ENDscript server. Nucleic Acids Res. 42, W320–324 (2014). 24753421 10.1093/nar/gku316 PMC4086106

[b29] Tang G. *et al.* EMAN2: an extensible image processing suite for electron microscopy. J. Struct. Biol. 157, 38–46 (2007). 16859925 10.1016/j.jsb.2006.05.009

[b30] Frank J. *et al.* SPIDER and WEB: processing and visualization of images in 3D electron microscopy and related fields. J. Struct. Biol. 116, 190–199 (1996). 8742743 10.1006/jsbi.1996.0030

[b31] Hohn M. *et al.* SPARX, a new environment for Cryo-EM image processing. J. Struct. Biol. 157, 47–55 (2007). 16931051 10.1016/j.jsb.2006.07.003

[b32] Nishiya Y., Tamura N. & Tamura T. Analysis of bacterial glucose dehydrogenase homologs from thermoacidophilic archaeon *Thermoplasma acidophilum*: finding and characterization of aldohexose dehydrogenase. Biosci. Biotechnol. Biochem. 68, 2451–2456 (2004). 15618614 10.1271/bbb.68.2451

